# Complete mitogenomes and phylogenetic relationships of *Haemaphysalis nepalensis* and *Haemaphysalis yeni*

**DOI:** 10.3389/fvets.2022.1007631

**Published:** 2022-11-02

**Authors:** Xin-yan Lu, Quan-fu Zhang, Dan-dan Jiang, Ya-fang Liu, Bin Chen, Shuang-ping Yang, Zong-ti Shao, Hang Jiang, Jian Wang, Yi-hao Fang, Chun-hong Du, Xing Yang

**Affiliations:** ^1^Integrated Laboratory of Pathogenic Biology, College of Preclinical Medicine, Dali University, Dali, China; ^2^Department of Digestion, First Affiliated Hospital of Chengdu Medical College, Chengdu, China; ^3^School of Public Health, Dali University, Dali, China; ^4^Yunnan Provincial Key Laboratory of Natural Epidemic Disease Prevention and Control Technology, Puer, China; ^5^Fu-gong Administration Bureau, Gaoligong Mountain National Nature Reserve, Yunnan, China

**Keywords:** ticks, *Haemaphysalis nepalensis*, *Haemaphysalis yeni*, mitogenome, genome annotation

## Abstract

The mitochondrial genome may include crucial data for understanding phylogenetic and molecular evolution. We sequenced the complete mitogenome of *Haemaphysalis nepalensis* and *Haemaphysalis yeni* for the first time. *H. nepalensis and H. yeni*'s complete mitogenomes were 14,720 and 14,895 bp in size, respectively, and both contained two ribosomal RNA (rRNA) genes, 22 transfer RNA (tRNA) genes, and 13 protein-coding genes (PCG). *Haemaphysalis nepalensis* have one control region (D-loop). The adenine + thymine concentration of the genomes of *H. nepalensis* and *H. yeni* was 77.75 and 78.41%, respectively. The codon use pattern and amino acid content of proteins were both observed to be affected by the AT bias. Genes in the mitogenome were organized and located in a comparable manner to previously known genes from *Haemaphysalis* ticks. Mitochondrial PCGs were used to perform phylogenetic relationships based on the Minimum Evolution (ME) approach using MEGA 7.0 software, the results reveal that *H. nepalensis* has tight links with *H. tibetensis, H. yeni* and *H. kolonini* share a sister group relationship, and that *H. nepalensis* and *H. yeni* belong to *Haemaphysalis*. The results of this study include the following: (i) discovered and supplied new tick records (*H. nepalensis*) for China, (ii) provided the first complete mitochondrial genome for *H. nepalensis* and *H. yeni* and revealed their phylogenetic relationships, and (iii) the features of the mitochondrial genome of *H. nepalensis* and *H. yeni* provided more genetic reference for Phylogeography, systematics, and population genetics of the *Haemaphysalis* species.

## Introduction

Ticks are obligatory ectoparasites of all vertebrate species and are blood-sucking arachnids. They can spread the broadest spectrum of zoonotic pathogens that lead to animal and human diseases, causing substantial financial damage to animal productivity (1). Tick populations are also growing as a result of climate change (2). Recently, numerous major tick-borne pathogens have been identified in ticks, such as *Babesia ovata, Chlamydiaceae bacteria, Rickettsia japonica, Anaplasma bovis, and Severe Fever with Thrombocytopenia Syndrome Bunyavirus*, have sparked increased interest in the field of public health (3). Despite the medical importance of ticks in the spread of Lyme disease, spotted fever group rickettsiosis, and other human diseases, the details of the entire mitochondrial genome are not well recognized, and the phylogenetic links are not established (4).

*Haemaphysalis nepalensis* (5) (Ixodidae) is an important tick that belongs to the Ixodidae family, Metastriata group, Herpetobia Canestrini subgenus. *H. nepalensis* has previously been discovered in India and Nepal. *H. nepalensis*, a parasite that affects people, sheep, and dzo, is most widely distributed in Tibet in China (5). India, Japan, the Philippines, Indonesia, Ceylon, Borneo, and China are the main distribution areas for *Haemaphysalis yeni* (6) (Ixodidae). In China, Fujian, Guangdong, Hainan, Hunan, Hubei, Shanxi, and Yunnan account for the majority of the records (7). Although the morphological characteristics of *H. nepalensis* and *H. yeni* have been illustrated and documented, it is unknown what their complete mitochondrial genome looks like (8). In recent years, tick bite reports on people have increased. The quality of human health continues to decline as a result of relapsing and persistent illness, long-term effects linked to tick-borne diseases, and even fatalities brought on by delayed or incorrect diagnosis. It is crucial to verify tick classification in order to combat disease (9).

The mitochondrion, a vital organelle in eukaryotes, contains a distinct genome from the cell nucleus. The mitogenome typically contains minimal levels of recombination, a simple structure, maternal inheritance, and fast evolution. It is therefore acknowledged as one of the most trustworthy and effective molecular tools for research on tick phylogenetic studies, species identification, and population structure (10). Complete mitochondrial genomes are normally double-stranded, with the length of circular nucleotides ranging between 14 and 19 kb, and consist of 13 PCGs: *cox1-cox3, nad1-nad6, atp8, atp6, nad4l*, and *cytb*, 22 tRNAs, two rRNAs, and D-loop. Researchers have used mitochondrial genomes as instructive molecular labels to investigate numerous evolutionary studies among animals (11). Recent advances in sequencing technology have made it easier than ever to reconstruct phylogenies using animal mitochondrial genomes in their entirety rather than just partial DNA sequences.

Using Illumina sequencing technology, we first sequenced and annotated the entire mitochondrial genomes of *H. nepalensis* and *H. yeni* in the current work, then we compared them to other *Haemaphysalis* mitochondrial genomes. Further study of phylogenetics, mitochondrial genomes, nuclear rRNA genes, and taxonomy revision of related *Haemaphysalis* species and the Ixodidae family may benefit from the information provided here (12).

## Materials and methods

### Samples and DNA extraction

The *H. nepalensis* adult specimens used in this study were procured in October 2020 from Deqin in the Yunnan Province of China (27°33′N, 98°3′E) (*n* = 2, female; *n* = 1, male), Diqing Tibetan Autonomous Prefecture. Adult samples of *H. yeni* were collected in March 2021 from High Li Gong Shan in the Nujiang Lisu Autonomous Prefecture of the Chinese Yunnan Province (26°34′N, 98°48′E) (*n* = 2, female; *n* = 1, male). Professor Chunhong Du examined the species' morphological identification using the essential diagnostic features (13). After collection, one adult female specimen was used for DNA extraction, and the remainder of the ticks were held as voucher specimens. The collected tick specimens were deposited at the Parasitological Museum of Dali University under the voucher numbers DLUP2010 and DLUP2103, respectively. The samples were maintained at −80°C and preserved in 95% alcohol before being utilized for DNA extraction. Single tick genomic DNA was extracted using DNAzol (Life Technologies, USA) following the manufacturer's instructions and stored for further processing (13).

### DNA amplification and sequencing

Two overlapping sets of primers were used to amplify the mitogenomes of the *H. nepalensis* and *H. yeni* species. The long-PCR primers were created using the *12S rRNA* and *cox1* genes of *Haemaphysalis bancrofti* (NC041076) and *Haemaphysalis japonica* (MG253031). The following PCR primers were employed:

HN1 (5′-CTCYAATTAAATTCTTTATRGAAT-3′, 5′-ATTAGGCTTGGTTGTATGAAWAA-3′);
HN2 (5′-TCTGTATTAAYTACAGCAATTTTAC-3′, 5′-CAAATWTTAAATTTAACACCCCAATTTTA-3′).
HY1 (5′-CTCTAGTTAATYTTGTGCCAGCAA-3′, 5′-AGCAACAGCGGTTATACAAWAAG-3′);
HY2 (5′-TCCGTATTAATTACTGCAATTCTAC-3′, 5′-CAACTTTAWAATGTAACACTCCAATCTTA-3′) (14).

The PCR was implemented in a 50 μl reaction mixture including 10 μl of 5X PrimeSTAR GXL Buffer (Takara, Japan), 1 μl of PrimeSTAR GXL DNA Polymerase (Takara, Japan), 4 μl of each primer, 4 μl of dNTPs, 4 μl of DNA template, and 23 μl of nuclease-free water. The PCR conditions used in the amplification procedure were as follows: initial denaturation at 95°C for 5 mins, followed by 45 cycles of denaturation (98°C for 10 s), annealing (68°C for 30 s), and extension (68°C for 10 mins), and a final extension was subjected to 68°C for 10 mins. The findings of the PCR were examined using 1.2 percent agarose gel electrophoresis stained with ethidium bromide (15). Libraries were sequenced on the Illumina HiSeq 2500 platform at Shanghai Biotechnology Co. Ltd. after the amplified PCR products had been purified.

### Gene annotation and sequence analysis

Data quality was evaluated from four indicators: single base quality of sequencing data, base content distribution, GC content distribution, and sequence base quality. The software used is FastQC (https://www.bioinformatics.babraham.ac.uk/projects/fastqc). AdapterRemoval software (v2.0) was used to remove the contamination of original data, and SOAPec software (v2.01) was used to correct the quality of data based on k-mer distribution. With the mitochondrial genome sequence of *H. kolonini* as a reference, the whole mitogenomes of *H. nepalensis* and *H. yeni* were extracted using online BLAST tools (https://blast.ncbi.nlm.nih.gov/Blast.cgi). SPAdesv3.9.0 (http://cab.spbu.ru/software/spades/) and A5-miseqv20150522 were utilized to compile high-quality next-generation sequencing data for *de novo* mitogenome construction (16). BLASTn (BLAST v2.2.31+) alignment was performed between the sequences with high sequencing depth and the NT Library in NCBI to pick out the mitochondrial sequences of each spliced result. The mitochondrial stitching results obtained by the above different software were analyzed by using MUMmer (v3.1) software for collinearity analysis to determine the position relationship between conting and to fill the gap of contigs. Pilon software (v1.18) was used to correct the results to obtain the final mitochondrial sequence. Using the MITOS web server (http://mitos.bioinf.uni-leipzig.de/), the mitochondrial genomes of *H. nepalensis* and *H. yeni* were annotated (17). The program tRNAscan-SE was used to forecast the secondary cloverleaf architectures of tRNAs (18). The skewness of the composition was estimated using the formulas GC-skew = [G – C]/[G + C] and AT-skew = [A – T]/[A + T]. The analysis of the nucleotide composition and relative synonymous codon usage was done using the software MEGA v7.0 and Geneious Prime (RSCU). The whole mitogenome annotation findings were submitted to NCBI in table (.tbl) format (19).

### Phylogenetic analysis

Based on the concatenated datasets of 13 PCGs from 40 ticks, including 34 Metastriata and six species of Prostriata, the phylogenetic connection was examined. The concatenated nucleotide sequence of the mitochondrial 13 PCGs was used to determine the evolutionary relationship using the ME approach in the software MEGA v.7.0 based on 1,000 bootstrapped datasets. The PCGs' multiple sequence alignments were conducted using the MUSCLE nucleotide mode. All places with incomplete or blank information were eliminated. Based on the Akaike information criterion (AIC), the GTR + G + I best model was selected as the best-fit replacement model for nucleotide phylogenetic connectivity (20). To examine the RSCU and nucleotide composition, MEGA program was utilized. A chronogram made with FigTree v1.4.2 was used to demonstrate the evolutionary relationships that emerged.

### Genome organization and nucleotide composition

**Figure 1:**
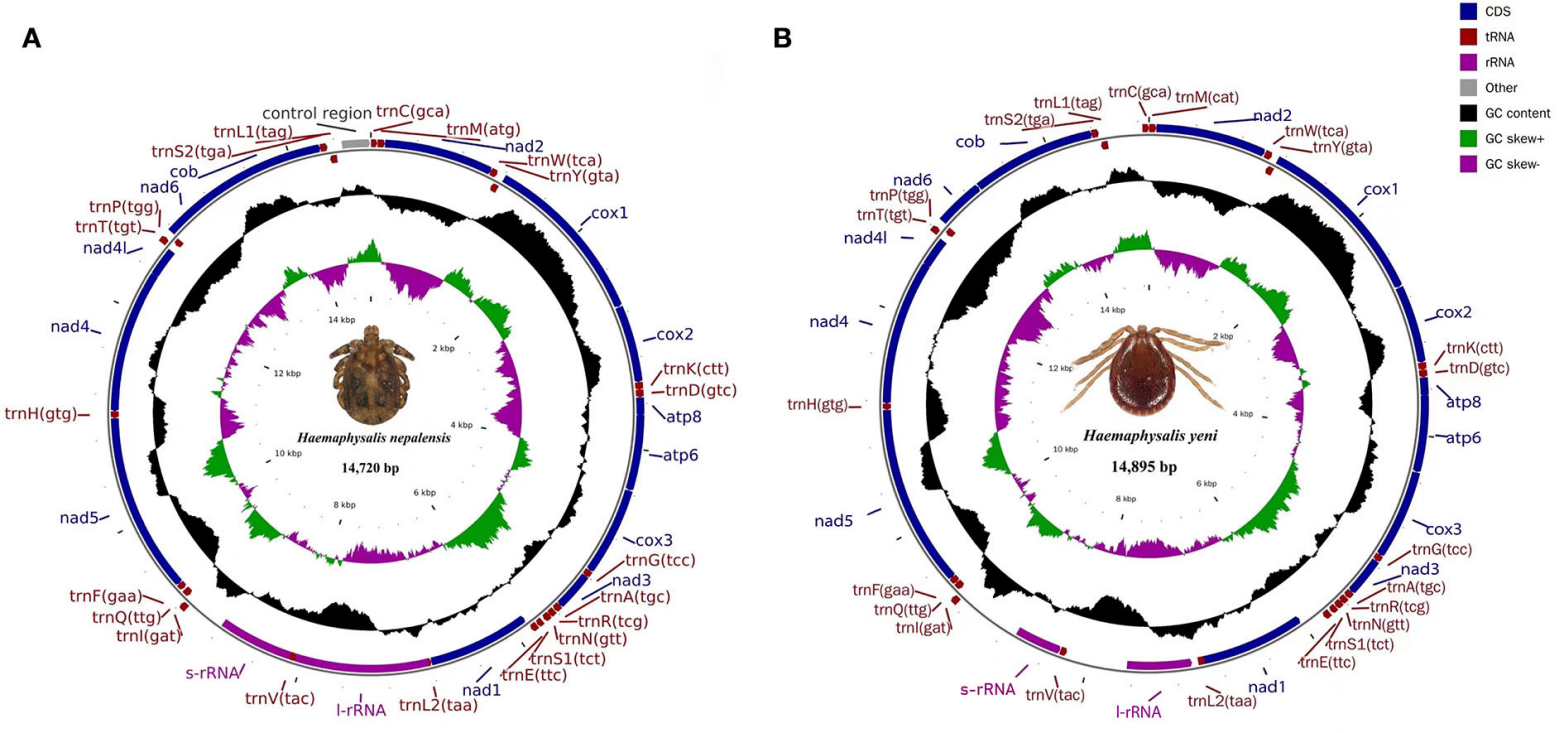
Mitochondrial genome map of **(A)** *H. nepalensis* and **(B)** *H. yeni*. Genes encoded in the forward direction are located on the outside of the ring, while those encoded in the reverse direction are located on the inside of the ring.

The entire mitogenomes of *H. nepalensis and H. yeni* used in this study were closed circular molecules with a size of 14,720 and 14,895 bp, respectively (Figure 1). The complete mitochondrial genome had 37 distinguishing genes, including 22 tRNAs, two rRNAs (*rrnL* and *rrnS*), 13 PCGs (*cox1-3, nad1-6, atp8, atp6, nad4l, and cob*), and *H. nepalensis* has one D-loop (Table 1). Under the accession numbers NC064124 and ON853615, the whole mitochondrial genomes of *H. nepalensis* and *H. yeni*, respectively, had been uploaded to GenBank. The mitochondrial genomes of *H. nepalensis* and *H. yeni* included the following nucleotide compositions: adenines = 38.46% (38.66%), thymines = 39.29% (39.74%), guanines = 9.51% (9.5%), and cytosines = 12.74% (12.28%). The complete mitochondrial genome of *H. nepalensis* and *H. yeni* were biased toward AT nucleotides (77.75 and 78.41%) (**Table 3**). Fourteen genes from both tick species are encoded on the majority (J) strand (Table 1). The *H. nepalensis* mitogenome includes 15 overlapping areas and intergenic nucleotides with a total length of 116 bp (range from 1 to 41 bp) and 464 bp (ranging from 2 to 334 bp). There were 14 overlapping areas in the mitochondrial genome of *H. yeni*, and the intergenic nucleotides were 49 base pairs long (ranging from 1 to 13 bp).

**Table 1:** Organization of the *H. nepalensis* and *H. yeni* mitochondrial genomes.

**Feature**	**Strand**	**Position**	**Length (bp)**	**Initiation codon**	**Stop codon**	**Anticodon**	**Intergenic nucleotide**
trnC	N	1–55/1–57	55/57			GCA	2/−1
trnM	N	58–122/57–119	65/63			ATG	
nad2	N	123–1,082/120–1,079	960/960	ATT/ATT	TAA/TAA		−2/5
trnW	N	1,081–1, 141/1,085–1,148	61/64			TCA	−2/1
trnY	J	1, 140–1,203/1,150–1,213	64/64			GTA	7/−8
cox1	N	1,211–2,734/1,206–2,744	1,524/1,539	ATA/ATT	TAA/TAA		4/4
cox2	N	2,739–3,414/2,749–3,423	676/675	ATG/ATG	T(AA)/TAA		
trnK	N	3,415–3,481/3,424–3,488	67/65			CTT	−1/−1
trnD	N	3,481–3,547/3,488–3,552	67/65			GTC	
atp8	N	3,548–3,706/3,553–3,714	159/162	ATC/ATT	TAA/TAA		−7/−7
atp6	N	3,700–4,365/3,708–4,373	666/666	ATG/ATG	TAA/TAA		3/3
cox3	N	4,369–5, 146/4,377–5,156	778/780	ATG/ATG	T(AA)/TAA		0/−1
trnG	N	5, 147–5,207/5,156–5,216	61/61			TCC	−3/0
nad3	N	5,205–5,546/5,217–5,558	342/342	ATA/ATT	TAG/TAA		−2/−1
trnA	N	5,545–5,605/5,558–5,619	61/62			TGC	4/3
trnR	N	5,610–5,669/5,623–5,682	60/60			TCG	−2/−2
trnN	N	5,668–5,728/5,681–5,741	61/61			GTT	19/2
trnS1	N	5,748–5,802/5,744–5,800	55/57			TCT	4/14
trnE	N	5,807–5,867/5,815–5,875	61/61			TTC	−7/184
nad1	J	5,861–6,802/6,060–7,010	942/951	ATT/ATT	TAA/TA(A)		/−13
trnL2	J	6,803–6,863/6,998–7,059	61/62			TAA	−41/56
rrnL	J	6,823–8,065/7,116–7,704	1,243/589				−10/556
trnV	J	8,056–8,117/8,261–8,321	62/61			TAC	−9/3
rrnS	J	8,109–8,793/8,325–8,744	685/420				334/575
trnI	N	9, 128–9, 192/9,320–9,385	65/66			GAT	
trnQ	J	9, 193–9,259/9,386–9,451	67/66			TTG	13/0
trnF	J	9,273–9,333/9,452–9,513	61/62			GAA	−1/−3
nad5	J	9,333–10,988/9,511–11,171	1,656/1,661	ATT/ATT	TAA/TA(A)		
trnH	J	10,989–11,052/11,172–11,230	64/59			GTG	0/−1
nad4	J	11,053–12,367/11,230–12,546	1,315/1,317	ATG/ATG	T(AA)/TAG		−7/−7
nad4l	J	12,361–12,636/12,540–12,815	276/276	ATG/ATG	TAA/TAA		2/2
trnT	N	12,639–12,701/12,818–12,879	63/62			TGT	0/−1
trnP	J	12,702–12,761/12,879–12,942	60/64			TGG	3/1
nad6	N	12,765–13,218/12,944–13,372	454/429	ATT/ATC	T(AA)/TAA		−21/3
cytb	N	13, 198–14,277/13,376–14,455	1,080/1,080	ATG/ATG	TAG/TAG		−1/−2
trnS2	N	14,277–14,339/14,454–14,515	63/62			TGA	6/−1
trnL1	J	14,346–14,411/14,515–14,579	66/65			TAG	56/315
OH	N	14,468–14,712/0	245/0				7/0

### PCGs and codon usage

Both *H. nepalensis* and *H. yeni* possessed 13 typical PCGs in their whole mitogenomes, including four cytochrome genes (*cytb and cox1-3*), two ATP genes (*atp6 and atp8*), seven NADP genes (*nad4l and nad1-6*) (Figure 1). The PCGs' respective regions were 10,828 bp and 10,837 bp in size. The PCGs of *H. nepalensis* began with ATA (*cox1 and nad3*), ATT (*nad1, nad2, nad5, nad6*), ATG (*cox3, atp6, cox2, nad4l, and nad4*), and ATC (*atp8*), also seven PCGs that were terminated by TAA (*cox1, nad2, atp8, atp6, nad1, and5, and nad4l*). The stop codon employed by the *cox2, cox3, nad4*, and *nad6* was a single T, whereas the *nad3* and *cytb* were terminated with TAG. The PCGs of *H. yeni* begin with ATT (*cox1, nad2, nad3, atp8, nad1, and nad5*), ATG (*cox3, atp6, cox2, nad4l, nad4, and cytb*), and only *nad6* with ATC. *Nad1* and *nad5* used incomplete termination codons that consisted of TA, while the majority of PCGs terminated with TAA (Table 2).

**Table 2:** List of the 40 tick species analyzed in this paper with their GenBank numbers.

**Species**	**Genus**	**Length (bp)**	**Genbank accession**
*Haemaphysalis nepalensis*	*Haemaphysalis*	14,720	NC064124
*Haemaphysalis yeni*	*Haemaphysalis*	14,895	ON853615
*Haemaphysalis concinna*	*Haemaphysalis*	14,675	NC034785
*Haemaphysalis formosensis*	*Haemaphysalis*	14,676	NC020334
*Haemaphysalis flava*	*Haemaphysalis*	14,686	NC005292
*Haemaphysalis hystricis*	*Haemaphysalis*	14,716	NC039765
*Haemaphysalis inermis*	*Haemaphysalis*	14,846	NC020335
*Haemaphysalis longicornis*	*Haemaphysalis*	14,718	NC037493
*Haemaphysalis doenitzi*	*Haemaphysalis*	14,671	NC062158
*Haemaphysalis sulcata*	*Haemaphysalis*	14,679	NC062063
*Haemaphysalis kolonini*	*Haemaphysalis*	14,948	MZ054209
*Haemaphysalis colasbelcouri*	*Haemaphysalis*	14,885	NC062164
*Haemaphysalis kitaokai*	*Haemaphysalis*	14,936	NC062161
*Haemaphysalis tibetensis*	*Haemaphysalis*	14,714	OM368296
*Haemaphysalis danieli*	*Haemaphysalis*	14,739	NC062065
*Dermacentor marginatus*	*Dermacentor*	15,178	NC062069
*Dermacentor reticulatus*	*Dermacentor*	14,806	MT478096
*Dermacentor nitens*	*Dermacentor*	14,839	NC023349
*Hyalomma asiaticum*	*Hyalomma*	14,723	NC053941
*Hyalomma truncatum*	*Hyalomma*	14,731	KY457529
*Hyalomma rufipes*	*Hyalomma*	14,761	NC061209
*Amblyomma marmoreum*	*Amblyomma*	14,677	KY457516
*Amblyomma testudinarium*	*Amblyomma*	14,760	MT029329
*Amblyomma geoemydae*	*Amblyomma*	14,780	MK814531
*Amblyomma sculptum*	*Amblyomma*	14,780	NC032369
*Ixodes tasmani*	*Ixodes*	15,227	NC041086
*Ixodes ovatus*	*Ixodes*	14,512	NC062061
*Ixodes ricinus*	*Ixodes*	14,566	NC018369
*Ixodes simplex*	*Ixodes*	14,556	NC062060
*Ixodes uriae*	*Ixodes*	15,053	NC006078
*Ixodes nipporensis*	*Ixodes*	14,505	NC058242
*Rhipicentor nuttalli*	*Rhipicentor*	14,779	NC039828
*Rhipicephalus australis*	*Rhipicephalus*	14,891	NC023348
*Rhipicephalus decoloratus*	*Rhipicephalus*	15,268	NC052828
*Rhipicephalus evertsi*	*Rhipicephalus*	14,739	KY457537
*Rhipicephalus simus*	*Rhipicephalus*	14,929	KJ739594
*Rhipicephalus maculatus*	*Rhipicephalus*	14,714	KY457539
*Rhipicephalus zambeziensis*	*Rhipicephalus*	14,691	KY457543
*Rhipicephalus turanicus*	*Rhipicephalus*	14,717	NC035946
*Archaeocroton sphenodonti*	*Archaeocroton*	14,772	NC017745

**Figure 2:**
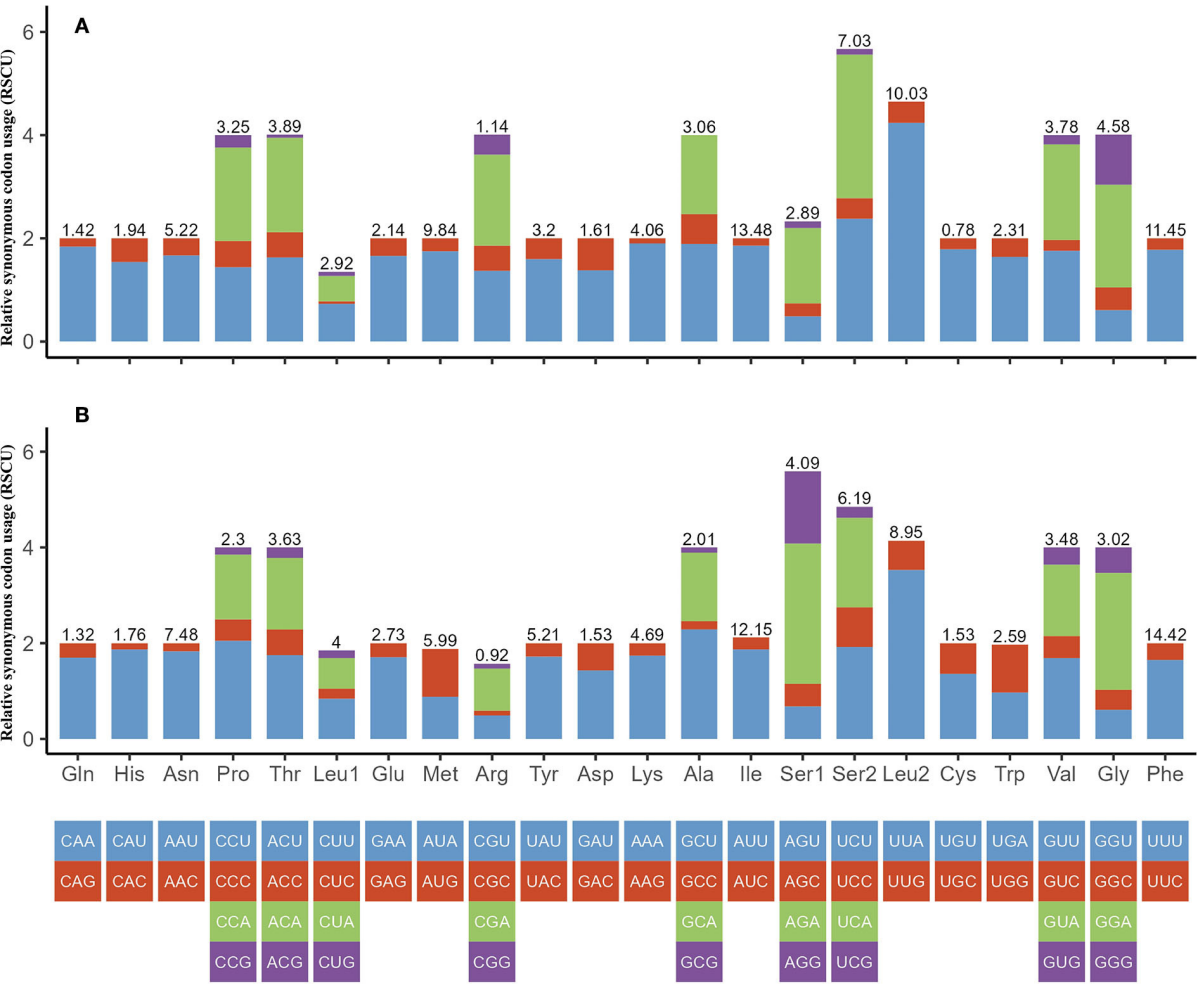
Relative synonymous codon usage (RSCU) of codons. **(A)** *H. nepalensis* and **(B)** *H. yeni*. The box below the bar chart represents all codons encoding each amino acid, and the height of the column above represents the sum of all RSCU values.

We investigated the RSCU and codon use patterns in the mitochondrial genomes of *H. nepalensis* and *H. yeni*. 3,405 amino acids in total were encoded by the mitochondrial genome of *H. nepalensis*. Leucine (16.18%) was the amino acid that was used the most, followed by phenylalanine (13.71%) and isoleucine (9.36%). The PCGs encoded a total of 3,474 amino acids of the *H. yeni* mitogenome; phenylalanine (14.42%), leucine (12.95%), and isoleucine (12.14%) were the most often utilized amino acids; arginine (0.9%) was the least frequently used amino acid, indicating the popularity of biasness toward AT content among the PCGs (Table 3; Figure 2).

**Table 3:** Composition and skewness of *H. nepalensis* and *H. yeni* mitogenome.

**Region**	**A%**	**C%**	**G%**	**T%**	**A+T%**	**G+C%**	**AT skew**	**GC skew**
Whole genome	38.46/38.65	12.74/12.28	9.51/9.31	39.29/39.74	77.75/78.39	22.25/21.59	−0.011/−0.014	−0.145/−0.138
nad2	38.54/37.18	11.15/10.52	5.31/5.63	45.00/46.67	83.54/83.85	16.46/16.15	−0.077/−0.113	−0.354/−0.303
cox1	31.04/31.50	16.08/15.07	14.63/14.10	38.25/39.31	69.29/70.81	30.71/29.17	−0.104/−0.110	−0.047/−0.033
cox2	36.83/36.14	16.57/15.41	10.06/9.78	36.54/38.67	73.37/74.81	26.63/25.19	0.004/−0.034	−0.244/−0.223
atp8	47.80/43.21	11.95/7.41	3.77/4.94	36.48/44.44	84.28/87.65	15.72/12.35	0.134/−0.014	−0.520/−0.200
atp6	32.13/33.62	13.06/13.06	9.01/8.71	45.80/44.59	77.93/78.21	22.07/21.77	−0.175/−0.140	−0.184/−0.200
cox3	31.23/28.72	14.01/13.21	11.18/11.79	43.57/46.28	74.81/75.00	25.19/25.00	−0.165/−0.234	−0.112/−0.056
nad3	32.75/29.53	7.89/10.53	9.65/9.65	49.71/50.29	82.46/79.82	17.54/20.18	−0.206/−0.260	0.100/−0.044
nad1	33.33/33.79	9.66/9.16	12.85/12.42	44.16/44.63	77.49/78.42	22.51/23.58	−0.140/−0.138	0.142/0.138
rrnL	41.35/38.88	7.32/9.00	11.26/15.79	40.06/36.33	81.42/75.21	18.58/24.79	0.016/0.034	0.212/0.274
rrnS	43.80/40.24	8.18/10.24	11.68/14.29	36.35/35.24	80.15/75.48	19.85/24.53	0.093/0.066	0.176/0.165
nad5	35.33/33.17	8.57/8.85	10.81/10.78	45.29/47.20	80.62/80.37	19.38/19.63	−0.124/−0.175	0.115/0.098
nad4	31.18/32.42	9.35/7.44	12.62/11.54	46.84/48.60	78.02/81.02	21.98/18.98	−0.201/−0.176	0.149/0.216
nad4l	34.78/35.51	5.80/3.26	9.78/10.51	49.64/50.72	84.42/86.23	15.58/13.77	−0.176/−0.176	0.256/0.527
nad6	39.21/41.03	9.47/8.86	4.85/5.83	46.48/44.29	85.68/85.32	14.32/14.69	−0.085/−0.038	−0.323/−0.206
cob	30.00/29.91	15.19/13.52	10.83/10.46	43.98/46.11	73.98/76.02	26.02/23.98	−0.189/−0.213	−0.167/−0.128
tRNA	40.00/39.50	8.69/9.00	11.68/11.70	39.64/39.80	79.64/79.30	20.37/20.70	0.005/−0.004	0.147/0.130
OH	31.84	16.33	16.33	35.51	67.35	32.65	−0.055	0.000

### A+T skewness and transfer RNAs

Positive AT and GC skew in the whole mitochondrial genomes of *H. nepalensis* and *H. yeni* indicates that bases A and G are less frequent than their comparable bases (Table 3). The mitogenomes of *H. nepalensis* and *H. yeni* had a set of 22 tRNAs, like the majority of mitochondrial genome DNA. The tRNAs of *H. nepalensis* are 1,370 bp long and range in size from 55 nucleotides (trnC and trnS1) to 67 nucleotides (trnK, trnD, and trnQ). *H. yeni*'s tRNAs were between 57 and 66 bp in length. The *tRNA-C, tRNA-F*, and *tRNA-S1* genes, as well as the 14 tRNAs encoded on the majority (J) strand, did not exhibit the normal cloverleaf structure.

### Phylogenetic analysis

**Figure 3:**
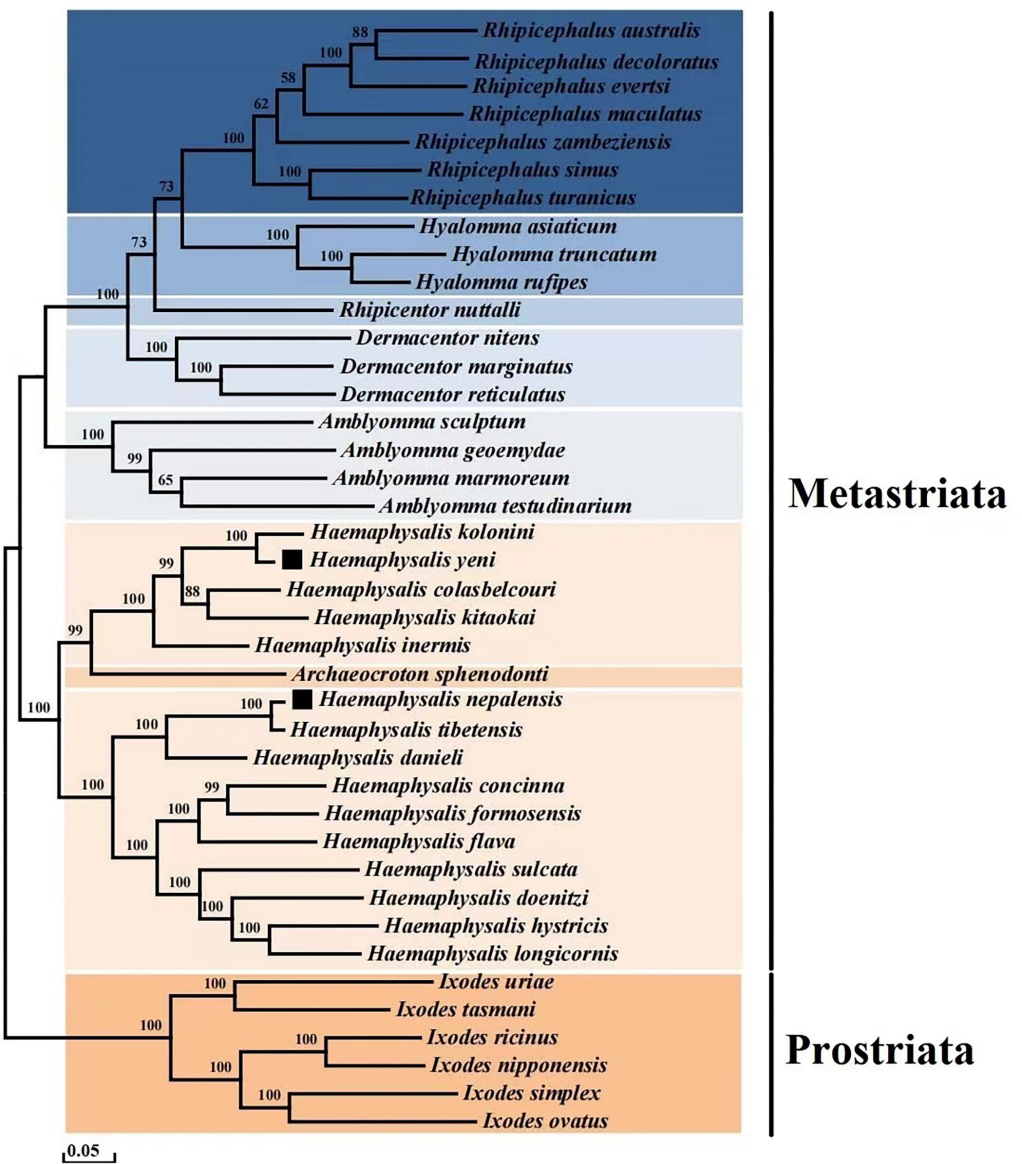
Phylogenetic analysis based on the nucleotide sequences of the 13 PCGs in the mitogenome. Each genus is represented by different colors.

To determine the phylogenetic tree of 40 Ixodida, the whole mitogenomes of *H. nepalensis* and *H. yeni* were further examined (Table 2). Using ME investigations in the context of the Maximum Composite Likelihood model, the topologies of the phylogenetic tree were examined based on the concatenated nucleotide sequences of 13 PCGs (Figure 3). The majority of the genera *Ixodes, Amblyomma, Dermacentor, Hyalomma*, and *Rhipicephalus* in the tree formed a monophyletic branch in the phylogenetic analyses, according to the ML analyses. *Haemaphysalis* species were paraphyletic, despite this. The sequences in the tree are divided into the Metastriata and Prostriata main branches. There is only one explicit *Ixodes* species in the Prostriata group. *H. nepalensis* and *H. tibetensis* are clustered together on one branch of the phylogenetic tree with a high nodal support value, and *H. yeni* and *H. kolonini* have a close phylogenetic relationship, indicating a sister group link between them. Furthermore, the phylogenetic relationship revealed that *H. nepalensis* and *H. yeni* were divided into various clades, yet they belonged to *Haemaphysalis* within Ixodida, correlating with other studies.

## Discussion

Similar to previously known tick mitochondrial genomes, a comparison of the mitochondrial genome sequences in the genus *Haemaphysalis* suggests that the D-loop is where the size shift is most prominent. The D-loop is the largest non-coding segment of the mitogenome and contains the major regulatory elements for its replication and expression. Furthermore, the high-level of intraspecific genetic variation found in the D-loop favors its use in population genetic studies of all kinds of organisms and phylogeographic analysis. Differences in the size of the complete mitogenome between species were driven by variation in the size of the control area, which in turn differed in both the size of different short repeat nucleotides and those replicated within it. In this study, most ticks in the *Haemaphysalis* genus have two D-loops. Nevertheless, *H. yeni* and *H. longicornis* have no D-loop, *H. nepalensis* and *H. hystricis* have one D-loop, and *H. colasbelcouri* and *H. kitaokai* have *three* D-loops. Because the D-loop lacks characteristic coding constraints, it accumulates indels, a variable number of tandem repeats, and base substitutions which are responsible for the widespread length differentiation found in the mtDNA molecule. Most of the tRNA secondary structures, the genome's codon use, and the gene makeup and quantity of *Haemaphysalis* are identical to other mitochondrial species that have been observed (21). The *tRNA-F* gene of *H. nepalensis*, however, differs from those of most of the genus *Haemaphysalis* in that it did not reveal the usual cloverleaf structure. The mitochondrial genomes' gene arrangement is comparable with that of other *Haemaphysalis*.

Through the creation of two new primer nucleotide sequences, next-generation sequencing technologies, and a brand-new long-range PCR amplification, we explored the mitogenomes. This will open a new way for study into *H. nepalensis* and *H. yeni* in the future. Population biology, behavior, phylogenetics studies, and tick ecology are all made easier by genetic information. Only the 16s rDNA partial sequences for *H. nepalensis* and *H. yeni* are currently available in the database. The mitochondrial partial sequencing can only offer relevant data, though. The entire mitochondrial genome can provide more sensitivity and resolution for analysis of the evolutionary relationships between closely related species as compared to partial mitochondrial sequence.

The current study shows that whereas *Haemaphysalis* species are paraphyletic, the majority of genera analyzed are monophyletic. According to the results of the phylogenetic analysis, there are two branches within the eight genera: one major clade contains a branch of *Ixodes* species that is quite explicit and monophyletic, and the other clade is made up of the genera *Haemaphysalis, Archaeocroton, Amblyomma, Dermacentor, Rhipicentor, Hyalomma, and Rhipicephalus*. Within the Metastriata, they are split into two branches, one of which has a sister-taxon (*Haemaphysalis* + *Archaeocroton*), and the other of which contains the genera *Amblyomma, Dermacentor, and Rhipicentor*, which are sisters to the sister-group genera *Hyalomma* and *Rhipicephalus*.

In addition, the clade (*H. nepalensis* + *H. tibetensis* + *H. danieli*) that includes our target species *H. nepalensis* grouped into one branch, and *H. yeni* and *H. kolonini* have strong links and a high nodal bootstrap support value.

The *H. nepalensis* and *H. tibetensis*, the *H. yeni* and *H. kolonini* genes are arranged in the same order and the PCG encoding of *H. nepalensis* is 11 bp less than the PCG encoding of *H. tibetensis*, and the PCG encoding of *H. yeni* is 35 bp more than the PCG encoding of *H. kolonini*. The percent identity of the *cox1* gene of these two groups species were 98.37 and 90.16%, respectively. The percent identity of the complete mitochondrial sequences were 98.14 and 92.75%, respectively. Species identification mainly depends on morphology and molecular studies. Although the two groups (*H. nepalensis* and *H. tibetensis, H. yeni* and *H. kolonini*) were phylogenetically very closely related in phylogenetic tree analysis, the morphological differences and percent identity are < 99%, which may lead to the formation of different species. The *H. tibetensis* and *H. nepalensis* species are now only known to have been found in Tibet alone. The phylogenetic analysis result demonstrates a close relationship between *H. nepalensis* and *H. tibetensis*, demonstrating the correctness of our sequencing findings and demonstrating the first distribution of *H. nepalensis* in Yunnan Province.

## Conclusion

In this study, we sequenced the entire mitochondrial genomes of H. *nepalensis* and *H. yeni*, measuring 14,720 base pairs (bp) and 14,895 base pairs (bp), respectively. These genomes contained 37 genes (13 PCGs, 22 tRNAs, and two rRNAs), which are typical of the *Haemaphysalis* mitochondrial genome. *H. nepalensis* had an extra D-loop. All PCGs began with the ATN codon, with ATT and ATG being the most frequent initiation codons. The *cox2, cox3, nad4*, and *nad6* of *H. nepalensis* and *nad1* and *nad5* of *H. yeni* had incomplete termination codons consisting of T or TA, and the other PCGs stop with the canonical TAG or TAA. The whole mitochondrial genomes of *H. nepalensis and H. yeni* had negative AT-skew and GC-skew, which is consistent with the majority of sequenced *Haemaphysalis*. Higher-level phylogenies might be provided by the whole mitochondrial genome. This study provides a crucial resource for better understanding the phylogenetics, molecular evolution, and population dynamics of these significant tick species.

## Data availability statement

The data presented in the study are deposited in the NCBI repository, accession number NC064124 and ON853615.

## Ethics statement

This study was approved by the Administration Committee of Experimental Animals, Dali University, First Affiliated Hospital of Chengdu Medical College, and Yunnan Provincial Key Laboratory of Natural Epidemic Disease Prevention and Control Technology.

## Author contributions

X-yL and Q-fZ conceived the study and wrote the manuscript. D-dJ, Y-fL, BC, and S-pY carried out the experiments and analyzed the data. Z-tS, HJ, and JW contributed to the collection of *Haemaphysalis nepalensis* and *Haemaphysalis yeni* and discussions. Y-hF, C-hD, and XY are responsible for the interpretation of experimental data, critical revision of important knowledge content, and final approval of the version to be published.

## Funding

This work was supported by the National Natural Science Foundation of China (Nos. 81760607 and U2002219), Yunnan Natural Science Foundation (2017FD139), and Scientific Research Fund of Yunnan Education Department (2022J0687).

## Conflict of interest

The authors declare that the research was conducted in the absence of any commercial or financial relationships that could be construed as a potential conflict of interest.

## Publisher's note

All claims expressed in this article are solely those of the authors and do not necessarily represent those of their affiliated organizations, or those of the publisher, the editors and the reviewers. Any product that may be evaluated in this article, or claim that may be made by its manufacturer, is not guaranteed or endorsed by the publisher.
